# Rapid Detection of SARS-CoV-2 Variants of Concern, Including B.1.1.28/P.1, British Columbia, Canada

**DOI:** 10.3201/eid2706.210532

**Published:** 2021-06

**Authors:** Nancy Matic, Christopher F. Lowe, Gordon Ritchie, Aleksandra Stefanovic, Tanya Lawson, Willson Jang, Matthew Young, Winnie Dong, Zabrina L. Brumme, Chanson J. Brumme, Victor Leung, Marc G. Romney

**Affiliations:** St. Paul’s Hospital, Vancouver, British Columbia, Canada (N. Matic, C.F. Lowe, G. Ritchie, A. Stefanovic, T. Lawson, W. Jang, M. Young, V. Leung, M.G. Romney);; University of British Columbia, Vancouver (N. Matic, C.F. Lowe, G. Ritchie, A. Stefanovic, C.J. Brumme, V. Leung, M.G. Romney);; British Columbia Centre for Excellence in HIV/AIDS, Vancouver (W. Dong, Z.L. Brumme, C.J. Brumme);; Simon Fraser University, Burnaby, British Columbia, Canada (Z.L. Brumme)

**Keywords:** 2019 novel coronavirus disease, coronavirus disease, COVID-19, severe acute respiratory syndrome coronavirus 2, SARS-CoV-2, viruses, respiratory infections, zoonoses, B.1.1.28/P.1, N501Y, spike, K417N, British Columbia, Canada

## Abstract

To screen all severe acute respiratory syndrome coronavirus 2–positive samples in Vancouver, British Columbia, Canada, and determine whether they represented variants of concern, we implemented a real-time reverse transcription PCR–based algorithm. We rapidly identified 77 samples with variants: 57 with B.1.1.7, 7 with B.1.351, and an epidemiologic cluster of 13 with B.1.1.28/P.1.

A robust surveillance system for early identification of severe acute respiratory syndrome coronavirus 2 (SARS-CoV-2) variants of concern (VOCs) is of critical public health value. VOCs have demonstrated in vitro evasion of antibody neutralization (*1*,*2*; W.F. Garcia-Beltran et al., unpub. data, https://www.medrxiv.org/content/10.1101/2021.02.14.21251704v1) and displayed potential for enhanced transmission because of mutations in the spike receptor binding domain (G. Nelson et al., unpub. data, https://www.biorxiv.org/content/10.1101/2021.01.13.426558v1; H. Liu et al., unpub. data, https://www.biorxiv.org/content/10.1101/2021.02.16.431305v1). Surveillance from the United Kingdom demonstrated a rapid increase in cases during September 2020, attributed to the B.1.1.7 variant, which has become predominant in several other countries (*3*). A commercial SARS-CoV-2 real-time reverse transcription PCR (rRT-PCR) demonstrated dropout of the small (S) gene on B.1.1.7 because of a deletion mutation within the spike protein (69/70). Subsequently, the European Centre for Disease Prevention and Control proposed use of a specific commercial assay with 3 targets as a surveillance strategy (*4*).

The B.1.1.28/P.1 variant is an emerging VOC and the predominant strain in certain regions of Brazil. Although uncommon in North America, it has now been detected across several continents. Re-infection of patients with SARS-CoV-2 immunity has raised concerns about resurgence (*5*). Unlike B.1.1.7, the B.1.1.28/P.1 variant does not possess the 69/70 deletion mutation, highlighting the need for a versatile VOC surveillance strategy.

Given the potential for VOCs to enhance transmission, increase deaths, and possibly evade natural or vaccine-induced immune responses, identifying cases of coronavirus disease (COVID-19) caused by VOCs and monitoring their prevalence is critical. We propose a rapid VOC surveillance strategy that uses multiple rRT-PCRs to screen all samples positive for SARS-CoV-2. This study was approved by the Providence Health Care/University of British Columbia and Simon Fraser University Research Ethics Boards (H20–01055).

## The Study

During January 26–March 1, 2021, the clinical virology laboratory at St. Paul’s Hospital, Vancouver, British Columbia, Canada, conducted VOC testing on nasopharyngeal swab and saliva/mouth rinse samples in which SARS-CoV-2 was detected at any cycle threshold (C_t_) value. SARS-CoV-2 detection was performed by using the LightMix SarbecoV E-gene plus EAV control assay (TIB Molbiol, https://www.tib-molbiol.de), with the MagNA Pure Compact or MagNA Pure 96 and LightCycler 480 or with the cobas SARS-CoV-2 Test (Roche Molecular Diagnostics, https://diagnostics.roche.com) on the cobas 6800. VOCs were detected with the VirSNiP SARS-CoV-2 Mutation Assays for strain surveillance (TIB Molbiol), targeting specific spike protein variations (N501Y, delHV69/70, K417N, E484K, V1176F). A laboratory-developed test was also developed for N501Y (501F-GCATGTAGAAGTTCAAAAGAAAGT; 501R-TCCTTTACAATCATATGGTTTCCA; 501YProbe FAM-CACT+T+ATGGTGTTGGTTACCAACCA-IABkFQ; 501NProbe Cy5-CACT+A+ATGGTGTTGGTTACCAACCA-IAbRQSp) and delHV69/70 (del6970F-TCAACTCAGGACTTGTTCTTAC; del6970R-TGGTAGGACAGGGTTATCAAAC; wtProbeHEX-TGCTAT+ACATG+TCTCTGGGACCA-IABkFQ; delProbe-TEX615-TGCTAT+CTCTG+GGACCAATG-IAbRQSp), in which + denotes locked nucleic acids. Samples were first screened for N501Y, and if detected, we tested subsequent targets to discriminate between the most prevalent VOC within the Vancouver community (B.1.1.7-delHV69/70 and B.1.351-K417N) and newly emerging VOC (B.1.1.28/P.1-V1176F).

For the first presumptive case caused by each VOC, we performed whole-genome sequencing (WGS) in-house on either the MinION (Oxford Nanopore Technologies, https://nanoporetech.com), using the ARTIC nCOV-2019 sequencing protocol V1 (https://www.protocols.io/view/ncov-2019-sequencing-protocol-bbmuik6w) by using V3 primers, or on an Illumina MiSeq (https://www.illumina.com) by using a modified ARTIC nCOV-2019 protocol. Accurate base calling of MinION was performed by using GUPPY 3.1.5 and FASTQ files analyzed with BugSeq (https://BugSeq.com). Illumina data were analyzed with the in-house bioinformatics pipeline MiCall (https://github.com/cfe-lab/MiCall). All presumptive VOCs were subsequently sent to a reference laboratory for confirmatory WGS.

During the study period, 31,833 clinical samples were tested for SARS-CoV-2, and results were positive for 2,618. Of these, 2,430 (92.8%) underwent testing for the 3 major VOC categories (B.1.1.7, B.1.351, B1.1.28/P.1); 1.6% (38/2,430) failed to amplify with the N501Y assay, of which 71.0% (27/38) were reported as indeterminate for SARS-CoV-2, reflecting late C_t_ values and presumably low viral loads. From the remaining 2,392 samples, 77 VOCs were identified (57-B.1.1.7, 7-B.1.351, and 13-B.1.1.28/P.1). N501Y was not detected in the remaining 2,315 (96.8%) samples, and they were not sent to the reference laboratory for WGS. The VirSNiP and laboratory-developed PCRs were concordant for all detected VOCs.

During the study period, VOC detection among diagnostic samples rapidly increased (Figure). Of note, identified VOCs included a large cluster of the B.1.1.28/P.1 variant not previously identified in British Columbia. All B.1.1.28/P.1 variants were initially suspected from the K417N assay, for which PCR products were identified at a lower melting temperature than expected. All suspected B.1.1.28/P.1 variants were confirmed when rescreened by using the V1176F target.

**Figure:**
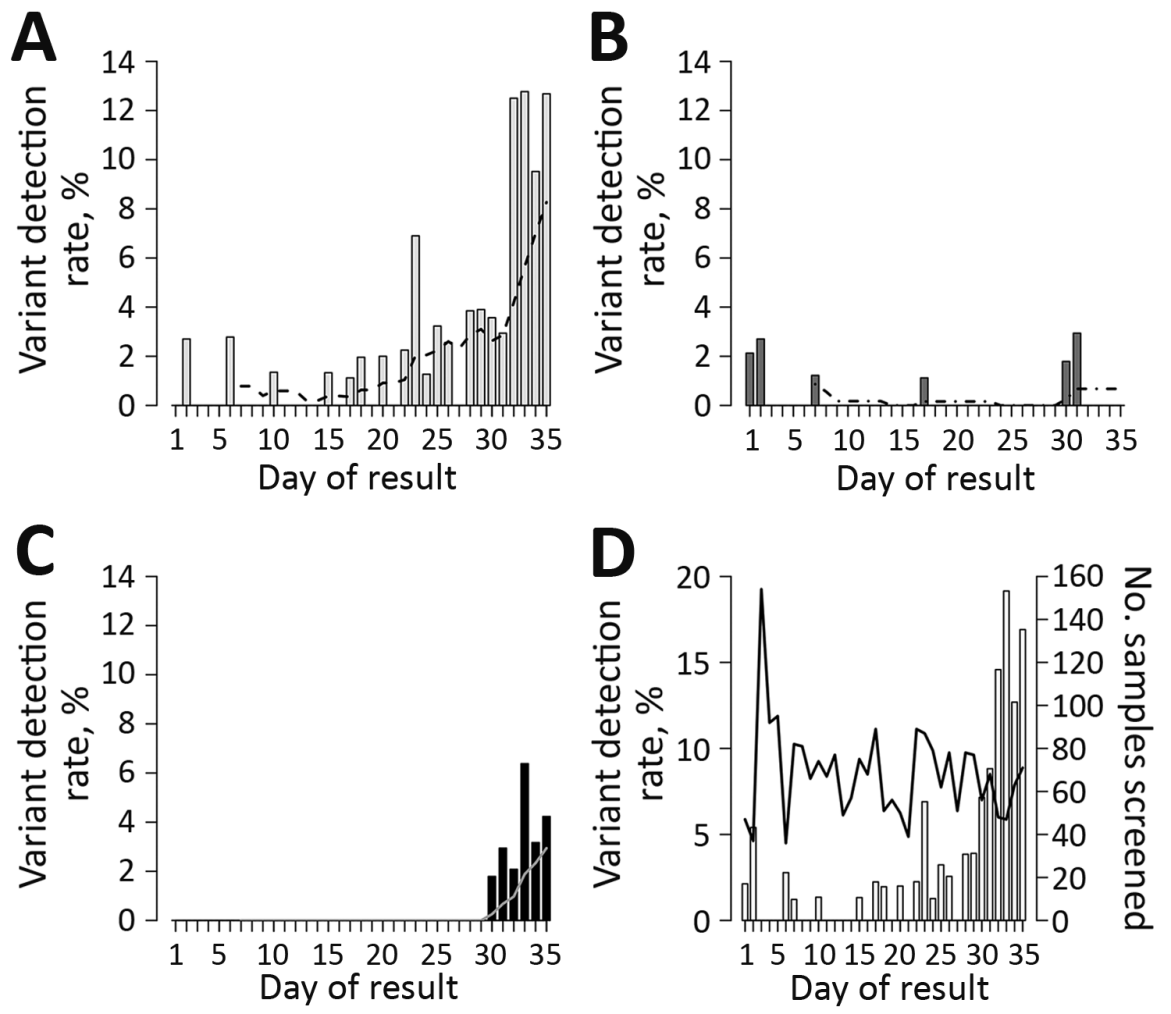
Rate of detection of severe acute respiratory syndrome coronavirus 2 variants of concern, by day of result, January 26–March 1, 2021, with 7-day moving average. A) B.1.1.7 (UK); dashed line indicates 7-day moving average. B) B.1.351 (South Africa); dashed line indicates 7-day moving average; C) B.1.1.28/P.1 (Brazil),;solid line indicates 7-day moving average; D) all variants of concern; solid line indicates number of samples screened.

The first presumptive B.1.1.28/P.1 variant identified was confirmed by in-house WGS, detecting the following S gene mutations (characteristic of B.1.1.28/P.1): L18F, T20N, P26S, D138Y, R190S, K417T, E484K, N501Y, D614G, H655Y, T1027I, and V1176F. At the time of publication, the reference laboratory had attempted WGS for 54/77 VOCs, of which 7 (12.9%) samples failed WGS, with original C_t_ values of 24–35. Of the 47 successfully sequenced samples, agreement with PCR was 100% (38-B.1.1.7, 3-B.1.351, and 6-B.1.1.28/P.1).

## Conclusions

Implementation of a PCR-based algorithm to detect VOCs has enabled our laboratory to rapidly detect new variants that are in the early stages of community transmission. Our protocol enabled detection of VOCs within 24 hours of COVID-19 diagnosis, a marked advantage over sequencing-based surveillance strategies. VOC positivity rate was 3.2%, but detection rates increased markedly over time, as might be expected with exponential growth observed in other countries.

Although B.1.1.28/P.1 had not been previously reported in our region, presence of this variant was suspected when K417N PCR products with a lower melting temperature than wild-type were observed, suggestive of a non-K417N substitution (e.g., K417T mutation in the B.1.1.28/P.1 variant) (VirSNiP SARS-CoV-2 Spike K417N package insert; TIB Molbiol, Berlin, Germany). Follow-up testing using the V1176F target supported the presumptive identification of B.1.1.28/P.1, which was subsequently confirmed by WGS. Testing all SARS-CoV-2–positive samples for VOCs enabled rapid detection of a discrete new B.1.1.28/P.1 cluster.

Although WGS has been the primary modality for VOC surveillance, universal sequencing of SARS-CoV-2–positive specimens is limited by both laboratory and bioinformatics capacity (*6*). Because of the volume of VOC testing and the limited capacity for high complexity WGS, turnaround times by WGS may be days to weeks. Since the onset of the COVID-19 pandemic, molecular diagnostics (i.e., PCR) have been increasingly adopted by laboratories to promptly identify SARS-CoV-2, and infrastructure has been established for this testing modality. A PCR-based algorithm for the molecular detection of VOCs could be rapidly adopted, providing almost real-time results to inform infection prevention and control and public health measures (*3*,*7**,**8*). PCR may also be more sensitive because WGS is challenging to perform on samples with low viral loads (C_t_ >30) (*9*). Compared with WGS, PCR screening enhanced sensitivity for VOC detection by >10%.

Although the most prevalent VOCs worldwide harbor N501Y, this mutation is not present in all variants (*10*). A PCR-based algorithm for identifying VOCs that use N501Y as the initial screening target must acknowledge this limitation. Given the rapid emergence of new variants, ongoing surveillance is key, and laboratories considering a PCR-based algorithm would need to adapt the algorithm as VOC prevalence changes. For example, our initial screening PCR targeted N501Y, but because of rising rates of B.1.1.7, we adjusted our laboratory-developed test to include N501Y and delHV69/70 in a duplexed assay.

PCR-based methods for rapid VOC detection should not replace broader VOC surveillance with WGS, which enables identification of non-N501Y VOCs and can characterize emerging mutations in known VOCs. This ability is critical for enabling laboratories to revise their PCR targets in an ongoing manner to keep pace with local VOC circulation.

In summary, our implementation of an rRT-PCR–based algorithm enabled identification of the most common VOCs to date (B.1.1.7, B.1.351, and B.1.1.28/P.1) within 24 hours. This method enables laboratories to perform VOC testing on all SARS-CoV-2–positive samples, enhancing VOC surveillance capacity to support near real-time decision making for interrupting transmission.
